# Preparedness of the community in facing disasters like earthquakes (Case: Cisarua, Indonesia)

**DOI:** 10.4102/jamba.v15i1.1438

**Published:** 2023-06-30

**Authors:** Totok D. Pamungkas, Silmi A. Aliyan, Ilham Nurfalah, Epon Ningrum, Enok Maryani

**Affiliations:** 1 Study Program of Geography Education, Faculty of Social Science Education, Universitas Pendidikan Indonesia, Bandung, Indonesia; 2 Study Program of Geographic Information Science, Faculty of Social Science Education, Universitas Pendidikan Indonesia, Bandung, Indonesia

**Keywords:** earthquake, community preparedness, knowledge, attitude, policies, emergency response plan, disaster warning systems, resource mobilisation

## Abstract

The potential for disaster in an area is based on the geographical characteristics of a place and the living factors of the surrounding community. Community preparedness must be implemented to minimise the impact of an earthquake. This study aimed to determine the level of community preparedness in facing earthquakes in Cisarua District, Indonesia, based on the results of earthquake hazard mapping. The research used the quantitative Analytical Hierarchy Process (AHP) for earthquake hazard mapping and disaster preparedness using questionnaires. The AHP parameters include earthquake acceleration, distance from the Lembang fault, rock type, soil type, land use, slope and population density. The sample for this study comprised 80 respondents from six villages with a relatively high level of vulnerability, namely the villages of Jambupida, Padaasih, Pasirhalang, Pasirlangu, Kertawangi and Tugumukti. Data collection was carried out through interviews and site surveys based on a questionnaire consisting of variables like knowledge and attitudes, policies, emergency response plans, disaster warning systems and resource mobilisation with a total of 80 respondents. The study results concluded that community preparedness was included in the unprepared category with a total score of 211. Aspects of kinship and kinship relations between residents greatly influenced community preparedness, and knowledge and attitudes were considered sufficient with a weight of 4.4. Increased public awareness of the potential for earthquake disasters must routinely be carried out through disaster emergency response outreach and training activities in addition to improving residents’ emergency response facilities.

**Contribution:**

The study findings highlight the village community’s earthquake disaster preparedness with the support of integrated spatial mapping of disaster vulnerability. The lack of awareness of the village community in earthquake disaster mitigation increases the level of disaster risk in their area.

## Introduction

**FIGURE 1:**
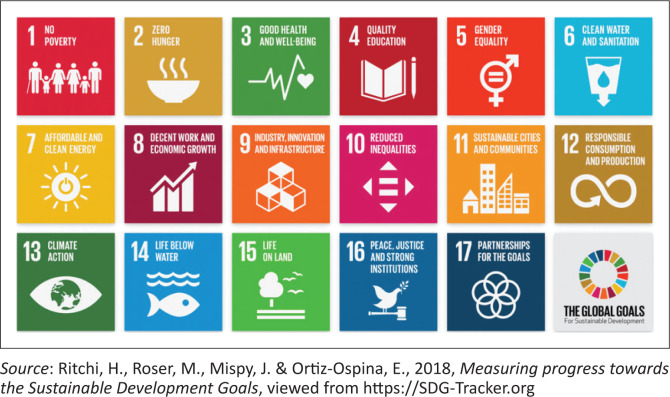
Sustainable development goals.

Starting from 17 goals of Sustainable Development as well as global and national commitments shown in Figure 1, namely development that supports the improvement of the economic welfare of the community, a development that maintains the sustainability of community social life a development that maintains environmental quality and outcome of justice and enforcement of good governance, can hold an improved quality of life from generation to generation. Implementation of goals as sustainable cities and settlements following the appropriate policies of the 2020–2024 National Medium-Term Development Plan, namely in developing disaster-resilient infrastructure and strengthening vital infrastructure, integrated management of disaster-prone areas, and restoration and conservation of watersheds.

The sustainable development goal concerned with natural disaster preparedness is Sustainable Development Goal number 11, namely ‘Building Inclusive, Safe, Disaster-Resilient and Sustainable Cities and Settlements’. This goal focuses on building cities and storage that can withstand the risks of natural hazards, protect residents from the effects of disasters and ensure access to basic facilities such as safe and sustainable air sanitation, sanitation and transportation. Other policies related to increasing disaster and climate resilience are implemented by strengthening the convergence between disaster risk reduction and climate change adaptation through disaster management strategies and increasing climate resilience. There is a need to create institutional mechanisms that are adaptable and amendable to meet the targets of Sustainable Development Goals at local levels (Triyanti et al. 2022). Implementing education and awareness in the community about climate change aligns with the points of the Sustainable Development Goals, namely increasing education, growing awareness and human and institutional capacity related to mitigation, adaptation, impact reduction and early warning of climate change. Public communication is essential as public awareness and participation in climate change adaptation and mitigation actions can be carried out by distributing information and campaigning to the public in a structured manner between institutions and the public.

### Location of a research area

Cisarua is a sub-district in West Bandung Regency, Indonesia. This district is about 9 km from the capital city of West Bandung regency to the northeast. The centre of government is in the village of Jambudipa. Cisarua is an area with great potential for agriculture and animal husbandry, with the main products being mushrooms, milk, secondary crops and vegetables. Cisarua has a relatively cold climate. Geographically, Cisarua District is the northern part of West Bandung Regency, located between 6° 3.73’ – 7° 1.031’ latitude and 107° 1.10’ – 107° 4.40’ east longitude, with an area of 95.56 km^2^ consisting of eight villages, namely Jambudipa, Kertawangi, Padasih, Pasirlangu, Pasirhalang, Cipada, Tugumukti and Sadangmekar (BPS Kabupaten Jawa Barat, 2022).

### Lembang Fault

Based on historical records, the Lembang Fault (PUSGEN 2017; Tjia 1968) is an escarpment that extends west-east, located north of Bandung City (Brahmantyo 2011; Rismawati 2019). This fault has continuity from the tip of the Cimandiri fault. Large earthquakes occurred along the Lembang fault in 1699, 1834 and 1900 (Visser 1922; Wichmann 1918). The slip rate of the Lembang fault reaches the range of 3 mm per year – 14 mm per year with sinistral shear movements (Meilano et al. 2012). The Lembang Fault is divided into six sections: Cimeta, Cipogor, Cihideng, Mount Batu, Cikapundung and Batu Bells (Daryono 2016).

The Cisarua area witnessed the last earthquake around the Lembang Fault on 22 July 2011, with a magnitude of 2.9 on the Richter Scale and on 30 August 2011, with a magnitude of 3.3 on the Richter Scale in Muril Village, Cisarua District which was severely impacted because of earthquake shocks (Basarah 2021; Nugraha et al. 2019; Rasmid 2014). Hundreds of houses were damaged, with light, moderate, to severe damage levels. From these two data, it is challenging to find a displacement process on the surface (Herman 2021). The approach of determining the earthquake’s hypocentre, relocation and analysis of the focus mechanism, as well as the influence of volcanism on the earthquake in the Lembang fault and surrounding settlements for further research in determining the micro-zoning of earthquake-prone areas and earthquake vulnerability in West Java (Daryono et al. 2018; Handayani et al. 2021; Pamungkas & Ningrum 2022; Tsasalatsa 2021) is very important to pursue. The characteristics of the social life of the people of Cisarua District in adapting to the surrounding environment are a very valuable knowledge capital and preparedness attitudes in dealing with disaster situations.

### Literacies community preparedness in surrounding West Java

Febrianti, Kuswanda and Winarni (2021) explained that in the analysis of the Sukamulya Village area, Langensari Village, Lembang District, it was noted that the area was earthquake-prone and landslide-prone as it was located on the Lembang fault line and close to the cliffs of Mount Batu, but did not explain the earthquake hazard mapping for the location in detail. The aim was to get the maximum sample of correspondents from the affected community. The research that was conducted focused more on the characteristics of the respondents through surveys and interviews, namely the level of physical (infrastructure), social and economic vulnerability of the people whose majority of jobs depended on nature with a high unemployment rate and a low level of education. Thus, the research focus is on community organisations such as counselling and forming community groups that are economically prepared to face disasters and reduce the consequential risk of earthquakes. Kastolani (2015) focused his research on counselling about community preparedness in Lembang District. The research area was selected based on the area close to the Lembang fault which has hilly topography and steep slopes with a potential for earthquakes, landslides and forest fires. This is different from Yunarto et al. (2019), previous research determined the research location based on the number and density of population as well as the distance from the location and the potential for earthquakes in the districts of Lembang, Parongpong and Ngamprah, West Bandung. The research results from the selection of knowledge and attitude index variables, emergency response plan variables with the almost-ready category and resource mobilisation with the not-ready category are the main parameters used to obtain the household preparedness index previously researched by Indrayani and Wasistiono (2021) who emphasised identifying the capabilities of facilities and infrastructure through focus group discussions with community protection organisations in West Java to increase community self-reliance in dealing with every disaster, looking at institutional aspects, human resources, effective policy implementation, finance, technical and leadership to improve disaster management capacity of an area both from pre-disaster to post-disaster. Community empowerment needs to be implemented as a protection unit locally. Romadona (2018) explained in his research using participatory action research methods with a qualitative approach and placing more emphasis on modelling school-based earthquake disaster risk reduction, as the area is threatened by potential earthquakes because of the active Lembang fault, West Bandung Regency. The Earthquake Alert School program was launched from the Mekarwangi Middle School and High School communities that are aligned with the conditions, needs and potential of the school environment. The initial conditions were compared with the final conditions of preparedness in the school community. The school community’s capacity is lacking and the high level of disaster risk is based only on the Lembang fault seismic data; the location of the school community is right above the fault.

It is about 100 m from the peak of Mount Batu Lembang. Meanwhile, earthquake hazard mapping and physical and socio-economic vulnerabilities were not visualised from the start to provide an overview of the extent to which an earthquake originating from the Lembang fault could impact the community and the school community in Lembang District. The study of Paramesti (2011) used similar instruments and parameters in analysing community preparedness for earthquakes influenced by the Cimandiri fault and the tsunami in the Teluk Pelabuhan Ratu area on the south coast of West Java for which people were unprepared. This research was not conducted to determine areas potentially prone to earthquakes and tsunamis at the beginning of the research in determining the sample of respondents. Mulyono, Elshap, and Kartika (2020) state the importance of effective disaster mitigation education in building public awareness about earthquake disasters, increasing knowledge about disasters and handling post-disaster situations, and strengthening awareness of caring for the surrounding environment. Meanwhile, Marlyono, Pasya, and Nandi (2016) are more focused on the importance of the community having literacy skills related to disaster information by showing the results of community preparedness in West Java through four primary indicators, namely the ability to identify and find information, evaluate information, organize and integrate information and utilize and communicate information effectively, legally and ethically. Research explains that the area of West Java region is prone to multiple disasters. Previous research tends to understand disasters from one aspect only, namely knowledge about the surrounding disaster, without involving aspects of policy, emergency response plans, disaster warnings, and resource mobilization. Furqon et al. (2018) conducted more specific research on participatory disaster risk assessment in the Cigadung sub-district, Bandung city, West Java, based on criteria like preparation (community profile), disaster history assessment, capacity and vulnerability assessment (community daily activities, household and community analysis), key respondent interviews and action planning. Based on interview data, it can be seen that most people are vulnerable to various disasters. Therefore the level of disaster awareness should be emphasized to the community. Nurjanah and Rezza (2021) created a community preparedness model in Bandung, West Java, emphasising social demographic factors, including attitudes, behavioural control, subjective norms, risk perception and behaviour and respondents’ experience dealing with disasters. This study shows that risk perception positively impacts disaster preparedness behaviour. Fitriyani, Emaliyawati and Mirwanti (2021) explain the sample’s readiness level in the student community of the Faculty of Development of the Padjadjaran University Campus in Garut Regency.

Although located in the same province of West Java, Garut has regional characteristics and geological structures that differ from the Cisarua-Lembang area, West Bandung Regency, where the earthquakes that frequently occur originate from the Garsela fault and the volcanic activity of Guntur and Papandayan mountains. The selection of sample locations was based on a map study from the Regional Disaster Management Agency for West Java Province. This study did not carry out the mapping directly and emphasized the readiness of student competencies as prospective health workers for emergency response in Garut Regency. Rosadi, Kadar and Istiadi (2020) conducted research that focused on observational correlations between disaster knowledge and environmental culture disaster preparedness behavior in a community of high school students in Karawang district, West Java. The research was not carried out by mapping and identifying the selection of research location samples directly. The research produced a positive correlation where knowledge of disaster and culture of caring for the environment increased, so students’ disaster preparedness behaviour also increased. Hastuti et al. (2020) used the multiple logistic regression analysis to test the influence of factors affecting community preparedness for earthquake disasters in Muruh Ganwarno Village, Klaten. Observations using the same variables are from LIPI-UNESCO/ISDR in 2016, with research results showing that knowledge is the factor that most significantly influences disaster-prone communities’ preparedness. The lesser the knowledge, the less prepared the community is to face disasters. Arif (2018) used the same questionnaire as the UNESCO-LIPI study for assessing community preparedness for earthquake disasters and reference data related to the earthquake news in Aceh that was experienced in the Takengon urban area, which resulted in fatalities being used as a sample for his research location. The research results indicated that the community was quite prepared but weak in terms of policies regarding rules and guidelines related to earthquake disaster preparedness and minimal emergency equipment. Maulida, Ocktadinata and Adhayanti (2022) focus on assessing earthquake and tsunami disaster preparedness for individuals or heads of households in the city of Cilegon during the coronavirus disease 2019 (COVID-19) pandemic without using disaster vulnerability mapping to find out the distribution of the sample, using the same questionnaire and variables, namely from LIPI-UNESCO/ISDR, where the results show that community preparedness is categorised as low or unprepared. This was because of the COVID-19 pandemic as the community was more focused on economic problems. The knowledge and attitude variables are strong categories, while resource mobilisation is categorised as lacking.

The novelty that distinguishes it from previous studies is the initial stage in selecting coverage areas that have a category of earthquake hazard value based on the Analytical Hierarchy Process (AHP) so that it effectively is an urgency to select respondents from the community from high to medium-referenced location area maps to be taken and used as samples, according to the needs. For reference, the questionnaire from LIPI-UNESCO/ISDR was modified as a Likert scale to facilitate field interview surveys. The research objective was to determine the level of community preparedness for earthquake disasters. The research also aimed to compare the level of community preparedness in various regions to assist policy-making in increasing community preparedness for earthquake disasters in West Java.

## Research methods

The research method used quantitative methods along with the AHP for earthquake hazard mapping. Analytical Hierarchy Process is used to eliminate and select the area’s most vulnerable location from the disaster vulnerability distribution map, which has a category value from high to low. Disaster preparedness using questionnaires uses statistical analysis. Data collection is carried out by distributing questionnaires to several respondents selected through the area categories of the AHP model disaster hazard map selected from the population of the village area that is the focus of the research. The validity and reliability of the data were tested in the study to ensure that the sample used was sufficiently representative of the population to be studied.

### Survey data collection

The earthquake hazard zoning map variable uses the following indicators: administrative boundaries, Lembang fault, land use, rock types, soil types, earthquake intensity, earthquake acceleration, slope and population density. Earthquake hazard mapping using the AHP method is largely determined by the weighting classification of each indicator (Ihsan et al. 2021; Serlia, Cahyono & Handayani 2021). The weighting classification consists of Peak Ground Acceleration (PGA) data which are the cause of damage to the surface in the area identified as earthquake-affected based on the Center for Volcanology and Geological Hazard Mitigation, Indonesia. For social studies, this study uses primary data-collection methods to determine community preparedness for disasters in the study area utilising direct surveys to locations, as well as through the distribution of questionnaires and structured interviews with community preparedness instruments for earthquake disasters derived from the main parameters sourced according to LIPI-UNESCO/ISDR (2006).

### Population and sample

**FIGURE 2:**
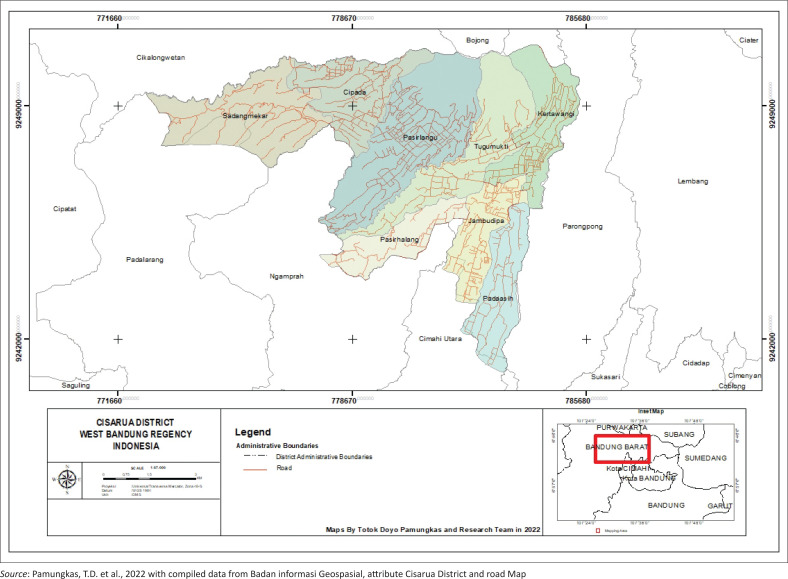
Location study area maps Cisarua District, West Java, Indonesia.

The population taken is the population of the area and residents around the Lembang fault area, namely Cisarua District, West Bandung Regency, Indonesia. The sample was determined through a physical study mapping to map the earthquake hazard zone around Cisarua District. The criteria for selecting community respondents in Cisarua District are based on the location of settlements in areas with high and moderate earthquake vulnerability levels. The data are obtained from Figure 2, the earthquake hazard mapping study results in the Cisarua sub-district, West Bandung Regency in 2022 using a scoring weight model through the AHP.

**FIGURE 3:**
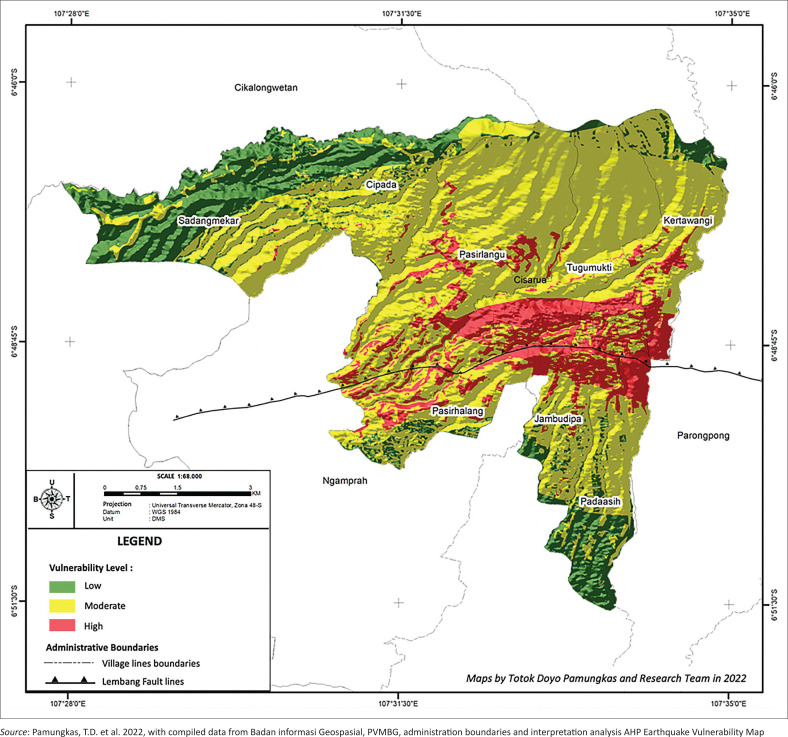
Earthquake Vulnerability Map in Cisarua District in 2022.

Figure 3 describes the earthquake hazard zone map around Cisarua District through AHP modelling, which is very detailed and efficient in helping to select sample areas in Cisarua District. Mapping uses the following earthquake hazard parameters: population density in each village, slope, geological rock, soil type, distance from the Lembang fault, PGA and land use. From the results of calculating the weight score for each parameter using AHP analysis, the area with the lowest vulnerability value is 1.803. The highest value is 3.03, with a low vulnerability classification (green) covering an area of 11 hectares or around 20.35%. In comparison, medium classification (yellow) covers an area of 3.5 hectares or around 64.8%, and for high classification (red) it covers an area of 0.8 hectares or around 14.85% of the total area of Cisarua District. The areas with a relatively low level of vulnerability, namely Cipada and Sadangmekar, can be eliminated to prioritise the urgency of interest in selecting areas that have a high and moderate level of vulnerability as research samples, namely, Jambupida, Padaasih, Pasirhalang, Pasirlangu, Kertawangi and Tugumukti villages. The total number of people living in the six villages is 64 920 people. Table 1 shows the population of six villages in Cisarua District in 2021.

**TABLE 1:** Total population of Cisarua District in 2021.

No	Village	Total population
1	Jambudipa	14 366
2	Padaasih	13 064
3	Pasirhalang	6 585
4	Pasirlangu	10 926
5	Kertawangi	13 032
6	Tugumukti	6 947

		64 920

*Source*: See www.disdukcapil.bandungbaratkab.go.id

To get the n-sample, the number of respondents is determined using the Slovin formula:


$$n=\frac{N}{(N.d^{2})+1}$$ [Eqn 1]

where the value of *n* = number of samples, *N* = total population and *d* = error rate. In this study, using an error rate of 11.5%, the number of respondents obtained with rounding results is 80, representing about six villages in Cisarua District, West Bandung Regency.

### Calculation of data validity test

Data collection was carried out by testing the validity of the Pearson Product Moment to determine the validity or suitability of the questionnaire used when measuring and obtaining research data. The results of observations in the *r* table obtained the value of the sample of respondents (*N*) = 80 of 0.220. Referring final validity test, it was found that all instruments ranging from knowledge and attitude variables, policies, emergency response plans, disaster warning systems and resource mobilisation consisting of 46 statements all resulted in the value of *r* arithmetic > *r* table and the significance value <0.05 so that the instrument data are valid.

### Calculation of statistical reliability data

The validated instrument used in the study is reliable first using the Cronbach Alpha reliability test (Cronbach 1951; Sujerweni 2014), which aims to determine whether the questionnaire is consistent if the repeated measurements were utilising questionnaires. From Table 2, it explains that each variable for earthquake disaster preparedness is seen based on descriptive statistical analysis both from the number of indicators, the mean, standard deviation and Cronbach’s alpha value.

**TABLE 2:** Descriptive statistical analysis of earthquake preparedness variables.

Variable	Number of indicator	Mean	Standard deviation	Cronbach’s alpha
Knowledge and attitude	10	33.39	8.09	0.868
Policy	5	11.45	4.63	0.784
Emergency response plan	17	42.84	12.93	0.896
Disaster warning system	6	12.66	5.24	0.775
Resource mobilisation	8	15.51	6.01	0.817

*Source:* Results of analysis of community preparedness earthquakes questionnaire data modified from (LIPI-UNESCO/ISDR, 2006) for descriptive statistical analysis using Microsoft Excel

The results for the knowledge and attitude variables with a total of 10 statements and the reliability value of Cronbach’s Alpha is 0.868. In the second variable, namely the policy with a total of five statements, the reliability value of Cronbach’s Alpha is 0.784. While the emergency response plan variable with a total of 17 statements, with the acquisition of Cronbach’s Alpha reliability value of 0.896. The fourth variable is the disaster warning system, with a total of six statements as the number of indicators, and the reliability value of Cronbach’s Alpha is 0.775. Moreover, the fifth variable is resource mobilisation, with eight statements. The reliability value of Cronbach’s Alpha is 0.817. So, the data are categorised as reliable because the value is >0.6 (Sujerweni 2016).

## Results and discussion

### Indicators of community preparedness for earthquake disasters

The indicators used to assess community preparedness come from the main parameters sourced from LIPIUNESCO/ISDR (2006). The interview instrument in the preparedness of the Cisarua District community in dealing with the earthquake disaster contains four variables consisting of knowledge and attitude variables with 10 indicators containing 10 statements, policy variables with five statement indicators, emergency response plan variables with 17 statement indicators, disaster warning system variables with six statement indicators and resource mobilisation variable with eight statement indicators. The instrument was modified as a Likert scale that was more effective in obtaining data and interpreting them quickly and efficiently. The assessment uses a five-choice Likert scale method (Sugiyono 2011). Giving the highest score (5) for the answer that strongly agrees; number (4) for the answer that agrees; number (3) for a neutral answer; number (2) for answers that do not agree and number (1) for strongly disagree answers.

The variable characteristics of the respondents that can be seen in Table 3 are an early stage in categorising some respondents regarding their general profile and level of preparedness for earthquake disasters. Based on the respondents’ education level in the field, around 61% had a high school education level. It can be concluded that most of the respondents had or learned while at school regarding disaster knowledge and risks. For the age variable in the 19–30 year category in Table 3, which means that the productive age is around 58%. The length of stay of respondents in the Cisarua area with the majority being more than 10–20 years is an important factor in disaster preparedness, in the sense that the experience of earthquakes that have occurred from time to time can be a lesson for them to anticipate and stay alert regarding earthquake disasters and knowing safe shelters and the location of the nearest health facility unit if they need assistance.

**TABLE 3:** Characteristics of respondents.

Variables	Categories	Frequencies (*n*)	Percentages (%)
Gender	Male	28	35
	Female	52	65
**Total**		80	100
Education level	Primary	27	34
	Secondary	49	61
	Higher	4	5
**Total**		80	100
Age	< 8 years old	9	11
	19–30 years old	46	58
	31–45 years old	3	4
	> 45 years old	22	28
**Total**		80	100
Occupation	Labour	8	10
	Salesman	10	13
	Employee	1	1
	Housewife	31	39
	Businessman	15	19
	Farmer	5	6
	Student	8	10
	Unemployed	2	3
**Total**		80	100
Length of stay	< 5 years	8	10
	From 5 to less than 10 years	5	6
	From 10 to less than 20 years	21	26
	> 20 years	46	58
**Total**		80	100

*Source:* Results of analysis of community preparedness questionnaire data in community prepareness earthquakes modified from (LIPI-UNESCO/ISDR, 2006) for the characteristics of respondents

### Class division and weighting per indicator

Class division and weighting were assessed from all indicators of each variable with a range of values from 0 to 100. The class division uses the Sturgess formula with the following equation: *K* = 1 + 3.3 Log (100) = 7.6, and the number of classes is rounded up to 8. While looking for the width of the interval class, the equation: *I* = (*R*/*K*), where *I* is the width of the interval, *R* is the range (highest value – lowest value) and *K* is the number of classes. So, from the equation, we get the width of the interval class *I* = (100−0)/8 = 12.5%. We can classify classes with the criteria and weight values as indicated in Table 4.

**TABLE 4:** Weighting of assessment classes per indicator.

Interval	Criteria	Weight
0–12.5	Not very good	1
12.6–25.1	Not good	2
25.2–37.7	Tend not to be good	3
37.8–50.3	Not enough	4
50.4–62.9	Enough	5
63–75.5	Tend to be good	6
75.6–88.1	Good	7
88.2–100	Very good	8

*Source:* Results of analysis of community preparedness earthquakes questionnaire data modified from LIPI-UNESCO/ISDR, 2006 for weighting of assessment classes per indicator using Microsoft Excel

### Community preparedness assessment for earthquake disasters

The assessment per indicator of community preparedness refers to the 2006 LIPI-UNESCO/ISDR instrument (Paramesti 2011), which is modified according to the type of disaster selected, namely only earthquake disasters can be seen in Table 5, resulting in a weighting score of 1–8 which will be accumulated against 46 other indicators to produce the final score of the assessment which will then be included in the interval class in Table 6, and the criteria can be determined based on the division of the Sturgess formula interval class and the width of the interval. The criteria range from ‘very unprepared’ to ‘very prepared’.

**TABLE 5:** Assessment of community preparedness indicators.

Variable	Percentage (%)	Total score	Average score	Criteria
Knowledge and attitude (10 indicators)	67	58	5.8	Enough
Policy (5 indicators)	47	22	4.4	Not enough
Emergency response plan (17 indicators)	50	79	4.6	Not enough
Disaster warning system (6 indicators)	42	23	3.8	Tend not to be good
Resource mobilisation (8 indicators)	39	29	3.6	Tend not to be good

**Total score**		**211**		

*Source:* Results of analysis of community preparedness earthquakes questionnaire data modified from LIPI-UNESCO/ISDR, 2006 for 5 assessment indicator results using Microsoft Excel

Note: Total indicators statement [*N* = 46]; Total respondent [*N* = 80].

**TABLE 6:** Interpretation of disaster preparedness scores.

Interval	Criteria
80–133	Very unprepared
134–187	Not ready
188–241	Not pretty ready
242–295	Pretty ready
296–349	Ready
350–400	Very prepared

*Source:* Results of analysis of community preparedness earthquakes questionnaire data modified from LIPI-UNESCO/ISDR, 2006 for interpretation of disaster preparedness scores

From Table 5, it can be seen that the preparedness of the people of Cisarua Subdistrict through knowledge and attitude variables has the highest percentage, namely 67% or with an average score of 5.8 this is in line with the research (Hastuti et al. 2020; Marlyono et al. 2016; Rosadi et al. 2020) with the criteria of being quite ready, and the lowest is resource mobilisation, which only reaches 39% or an average score of 3.6 tends to be not Good. The other three variables have a percentage less than or equal to 50%: policy, emergency response plan and disaster warning system.

Interpreting intervals from the value of earthquake preparedness is carried out using the Sturgess formula based on the sum of each earthquake preparedness indicator score. From Table 4, with a weight range of 1–8 and the number of indicators as much as *N* = 46, the highest possible value obtained using a Likert scale (5 choices) for 80 respondents is 400.The lowest value is 80. Using the Sturgess formula, we recalculate the class interval where

*K* = 1 + 3.3 Log (*N*) so that *K* = 1 + 3.3 Log (46) = 6.4 is rounded to six classes. For the width of the interval, *I* = (*R*/*K*) = ((400−80)/6) = 53. The percentage value is obtained by comparing the total value of each indicator in one variable to the maximum value of 400.

Table 6 shows the result of the calculation of the interval class with the criteria ‘very unprepared’ to ‘very prepared’.

The framework model for community preparedness for earthquake disasters carried out in this study includes the initial stages of mapping earthquake hazards, followed by the data-collection stage of questionnaire interviews with village residents as samples and continued at the stage of interpretation analysis to determine disaster preparedness scores. Mapping the distribution of disaster vulnerability in the Cisarua District area is very important. It helps to determine which villages need priority as samples and local government anticipation of increasing community capacity in dealing with disasters in six villages comprising Jambudipa, Padaasih, Pasirhalang, Pasirlangu, Kertawangi and Tugumukti with moderate to high vulnerability levels. This has never been done before in the early stages of selective and detailed sampling of area data. Meanwhile, the results of the disaster preparedness score from Table 6 based on the disaster preparedness variable in the six villages show a value of 211 with the criteria of not pretty ready compared to other areas that were more prepared or quite prepared in previous community preparedness research in West Java and its surroundings. A factor of the closeness of the respondent’s relatives to the family is considered to be good, and this becomes a strength in preparedness for caring and mutual protection, especially for the closest family in the event of a disaster. The role of the local government in socialising about disaster rescue actions and establishing a disaster post to help the community, especially with logistical needs in the field is also important. Existing evacuation routes usually are not noticed even though they have signs and are provided with road feasibility to indicate that people can pass during an earthquake. Earthquake detection equipment needs to be expanded to determine the intensity and epicentre of the earthquake, especially in areas prone to earthquakes.

## Conclusion

Based on the results of the study, it can be concluded that earthquake hazard mapping helps to determine the location of areas that are of concern to the government in the preparedness of the people of Cisarua District for earthquake disasters, especially the distance of settlements with medium and high levels of vulnerability whose radius is close to the Lembang fault. The preparedness of the people of Cisarua District who deal with earthquake disasters is included in the criteria of ‘not pretty ready’ with enough criteria for aspects of knowledge and attitudes. Although enough, aspects such as emergency response plans and disaster warning systems also require serious attention, because results tend to be not good. The kinship and closeness of relatives is a substantial factor in community cooperation. Local and central governments need to provide equipment for early detection of earthquakes, such as sirens, and ensure evacuation routes, disaster posts, and other emergency equipment to deal with disaster emergencies. Socialisation and training activities are critical in increasing awareness in the community regarding earthquake disaster preparedness and need to be carried out regularly, especially in areas prone to earthquake disasters.
